# Galectin 3-binding protein suppresses PRRSV replication via Cullin3-mediated ubiquitination degradation of non-structural protein 12

**DOI:** 10.1128/jvi.01083-25

**Published:** 2025-10-15

**Authors:** Xinrong Wang, Wenli Zhang, Juan Zhang, Rui Li, Longxiang Zhang, Nan Yan, Junhai Zhu, Lizhi Fu, Yue Wang

**Affiliations:** 1 College of Veterinary Medicine, Southwest University 26463, Chongqing, China; 2 Veterinary Medicine and Pharmaceuticals Research Institute, ChongQing Academy of Animal Sciences 580592, Chongqing, China; 3 National Center of Technology Innovation for Pigs, Chongqing, China; University of Michigan Medical School, Ann Arbor, Michigan, USA

**Keywords:** PRRSV, LGALS3BP, Nsp12, ubiquitination, antiviral activity

## Abstract

Porcine reproductive and respiratory syndrome virus (PRRSV) poses a major threat to the global swine industry, yet effective antiviral strategies remain limited. This study identifies galectin 3-binding protein (LGALS3BP) as a critical host factor inhibiting PRRSV infection through targeting the viral conserved non-structural protein 12 (nsp12), a key component of the viral replication-transcription complex. Overexpression of LGALS3BP significantly suppressed PRRSV replication, while its knockdown enhanced viral replication. Mechanistically, LGALS3BP recruits the Cullin3 E3 ubiquitin ligase via its BACK domain to mediate the ubiquitination of nsp12 at lysine residue 91, leading to proteasomal degradation. This process disrupts nsp12-dependent synthesis of viral subgenomic RNA, thereby disrupting replication. Additionally, LGALS3BP enhances antiviral innate immunity by upregulating interferon (IFN)-β and IFN-stimulated genes (ISGs). The antiviral effect of LGALS3BP is conserved across diverse PRRSV strains, highlighting its broad-spectrum potential. These findings reveal a dual mechanism whereby LGALS3BP restricts PRRSV through direct degradation of a critical viral enzyme and modulation of host immune responses, highlighting LGALS3BP as a promising therapeutic avenue for PRRSV control.

**IMPORTANCE:**

Porcine reproductive and respiratory syndrome virus (PRRSV) remains a major challenge to global swine production due to its genetic diversity, rapid mutation rate, and ability to evade host immunity. The nsp12 is highly conserved across PRRSV strains and plays a crucial role in viral RNA synthesis. This study identifies LGALS3BP as a critical host factor that inhibits PRRSV infection by targeting nsp12 via the ubiquitin-proteasome pathway. By uncovering this novel antiviral mechanism, the research highlights LGALS3BP as a promising therapeutic target for PRRSV control. Moreover, it contributes to our understanding of how host factors modulate viral replication and immunity, opening new avenues for developing host-targeted antiviral strategies. These findings have the potential to mitigate PRRSV-driven economic losses and improve swine health worldwide.

## INTRODUCTION

Porcine reproductive and respiratory syndrome virus (PRRSV), a member of the *Nidovirales* order and *Arteriviridae* family, is one of the most economically devastating pathogens in the global swine industry (1). Since its first identification in the late 1980s, PRRSV has caused reproductive failure in sows and respiratory diseases in piglets, with annual economic impacts exceeding billions of dollars worldwide (2, 3). PRRSV strains have been reclassified into two species: *Betaarterivirus suid 1* (PRRSV-1) and *Betaarterivirus suid 2* (PRRSV-2) by the International Committee on Taxonomy of Viruses (ICTV) (4). PRRSV presents significant genetic variations due to its high-frequency genome mutations, insertions/deletions, and recombination, making epidemic control a challenge (5). In China, classic PRRSV strains, such as CH-1a and BJ-4, were first identified after 1995. The highly pathogenic PRRSV variant (HP-PRRSV), including strains JXA1 and HuN4, emerged in 2006 (6, 7). Recent surveillance indicates that HP-PRRSV-like and NADC30-like strains are now the dominant variants, with increasing detection rates (8).

PRRSV has a single-stranded, positive-sense RNA genome of approximately 15 kb, encoding at least 10 open reading frames (ORFs). ORF1a and ORF1b are translated into large polyproteins that undergo autocleavage to generate 16 non-structural proteins (nsp1α, nsp1β, nsp2N, nsp2TF, nsp2-nsp6, nsp7α, nsp7β, and nsp8-nsp12) (9, 10). The replication and transcription complexes (RTCs), composed of viral and host proteins, are crucial for PRRSV replication (11, 12). ORF1b, the most conserved region of the arterivirus genome, encodes nsp9-12, which is a key component of the RTC (13). Among them, nsp12 was initially uncharacterized but is known to regulate subgenomic mRNA synthesis (+sgmRNA and −sgmRNA) without affecting minus-strand genomic RNA (−gRNA) synthesis (14).

Galectin 3-binding protein (LGALS3BP), also known as 90K or Mac-2BP, is a highly glycosylated, disulfide-linked oligomeric protein in the scavenger receptor cysteine-rich (SRCR) superfamily (15). Its multi-domain structure, comprising SRCR, BTB/POZ, and BACK domains, enables interactions with extracellular matrix components and contributes to immune regulation, cell adhesion, and tumor progression (16, 17). Recent studies report that LGALS3BP is upregulated during viral infections, such as HIV and herpesvirus, where it acts as a modulator of antiviral immunity (18, 19). In our previous study (20, 21), PRRSV infection could upregulate multiple host cellular genes, including LGALS3BP. However, its antiviral function in PRRSV remains unclear. This study investigates the role of LGALS3BP in PRRSV infection.

## RESULTS

### PRRSV infection induces LGALS3BP expression

**Fig 1:**
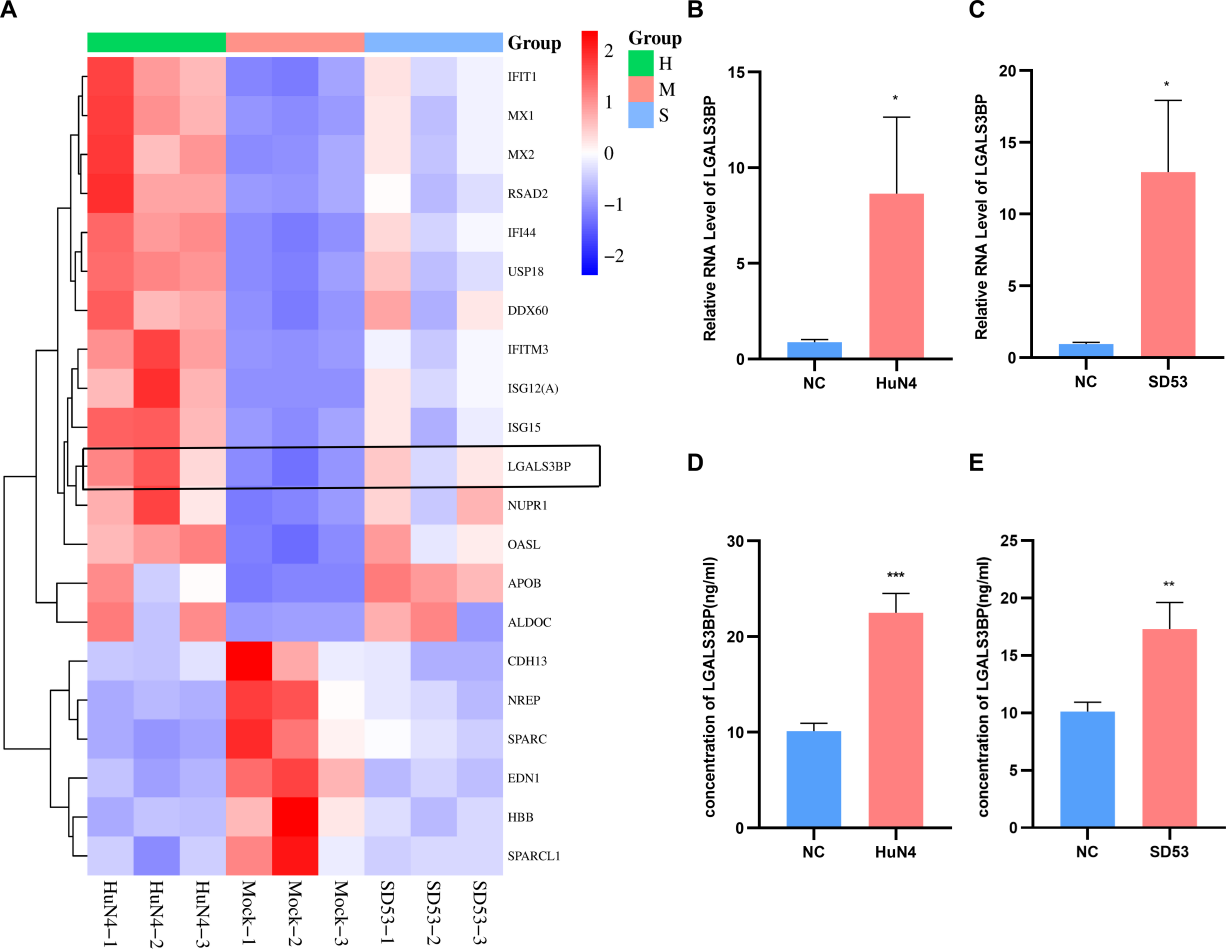
PRRSV infection induces the upregulation of LGALS3BP expression in the lungs. (**A**) Heatmaps show the core genes of the PPI network in the lungs of piglets infected with HuN4 and SD53. Samples are represented on the horizontal axis, and genes are represented on the vertical axis; red indicates high expression, and blue indicates low expression. (**B and C**) RT-qPCR analysis of *LGALS3BP* mRNA expression in lung homogenates at 3 dpi. Lung tissues of pigs infected with PRRSV-HuN4 (**B**) or PRRSV-SD53 (**C**) were collected to assess *LGALS3BP* mRNA levels by RT-qPCR. (**D and E**) ELISA analysis of LGALS3BP protein levels in lung tissues from pigs infected with PRRSV-HuN4 (**D**) or PRRSV-SD53 (**E**). Data are presented as the mean ± SD from three independent experiments. *, *P* < 0.05; **, *P* < 0.01; ***, *P* < 0.001. The *P* value was calculated using a Student’s *t*-test (two-tailed).

Based on our previous RNA-seq results (20, 21), LGALS3BP was significantly upregulated in response to both HuN4 and SD53 infections at 3 days post-infection (dpi), as shown in a heatmap (Fig. 1A). We then verified the *LGALS3BP* mRNA expression in lung homogenates. Reverse transcription quantitative PCR (RT-qPCR) analysis revealed that the *LGALS3BP* mRNA levels were significantly higher in pigs infected with either the HP-PRRSV strain HuN4 or NADC30-like strain SD53 compared with controls (Fig. 1B and C). To further validate this upregulation at protein levels, enzyme-linked immunosorbent assay (ELISA) was performed on lung homogenates, revealing a significant increase in LGALS3BP protein following infection with both strains (Fig. 1D and E). These data suggest that the upregulation of LGALS3BP may be part of the host’s antiviral response to PRRSV infection.

### LGALS3BP suppresses PRRSV replication

**Fig 2:**
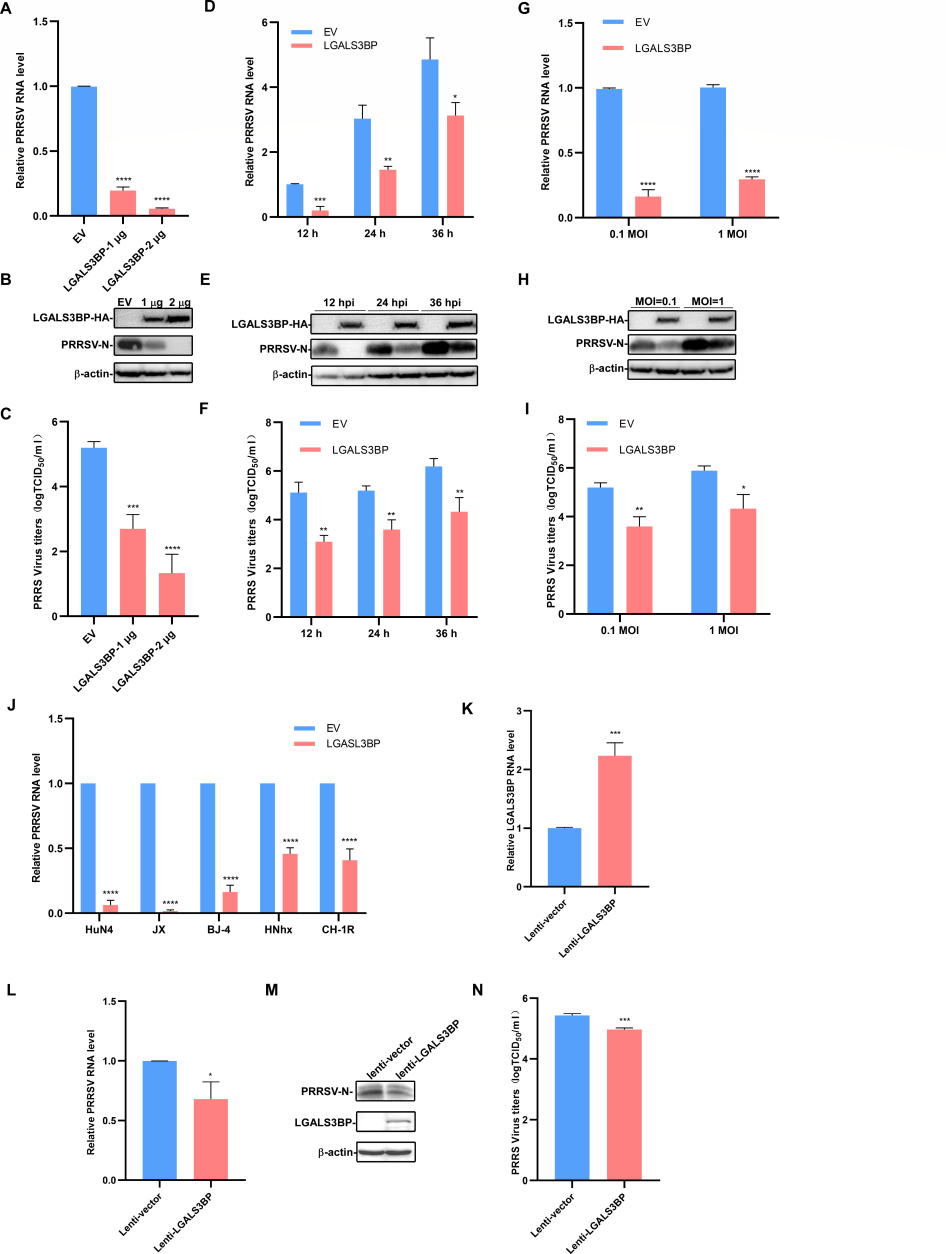
LGALS3BP overexpression suppresses PRRSV replication. (**A–C**) Marc-145 cells were transfected with EV or different doses of LGALS3BP, and then infected with PRRSV HuN4 (MOI = 0.1) for 24 h. The cell lysates and culture supernatants were collected to analyze PRRSV N protein expression and viral titers with RT-qPCR (**A**), Western blot (**B**), and TCID_50_ assay (**C**). (**D–F**) Marc-145 cells were transfected with EV or LGALS3BP and then infected with PRRSV (MOI = 0.1) for 12, 24, and 36 h. The cell lysates and culture supernatants were collected to analyze PRRSV N protein expression and viral titers with RT-qPCR (**D**), Western blot (**E**), and TCID_50_ assay (**F**). (**G–I**) Marc-145 cells were transfected with EV or LGALS3BP and then infected with PRRSV at MOI = 0.1 or 1 for 24 h. PRRSV N protein expression and viral titers were assessed with RT-qPCR (**G**), Western blot (**H**), and TCID_50_ assay (**I**). (**J**) Marc-145 cells were transfected with LGALS3BP or EV for 24  h and then infected with the indicated PRRSV strains at 0.1 MOI for an additional 24 h. The cell lysates were collected to analyze PRRSV RNA levels. (**K–N**) PAMs were incubated with LGALS3BP lentivirus mixed with polybrene for 12 h and then infected with PRRSV for 24 h. The cell lysates and culture supernatants were collected to analyze *LGALS3BP* RNA levels (**K**), PRRSV RNA levels (**L**), PRRSV protein expression (**M**), and viral titers (**N**). Data are represented as the mean ± SD from three independent experiments. Statistical significance was determined by one-way ANOVA (*, *P* < 0.05; **, *P* < 0.01; ***, *P* < 0.001; ****, *P* < 0.0001).

To assess the role of LGALS3BP in PRRSV infection, we constructed a plasmid encoding LGALS3BP. Marc-145 cells were transfected with either empty vector (EV) or LGALS3BP plasmids for 24 h, followed by infection with 0.1 multiplicity of infection (MOI) PRRSV HuN4 for an additional 24 h. RT-qPCR analysis revealed significant, dose-dependent reduction in PRRSV mRNA levels with LGALS3BP overexpression (Fig. 2A). Western blot analysis confirmed this, as LGALS3BP suppressed PRRSV-N protein levels in cells treated with one or 2 µg LGALS3BP plasmids, seen via weaker band than EV controls (Fig. 2B). Additionally, LGALS3BP-overexpressed cells had significantly lower viral titers (Fig. 2C). To further evaluate the effect of LGALS3BP on PRRSV replication, we examined viral RNA, protein, and titers at different time points and MOIs. The results showed significant reductions at 12, 24, and 36 h post-infection (hpi) (Fig. 2D through F). Moreover, LGALS3BP overexpression resulted in a significant decrease in PRRSV replication across different MOIs (Fig. 2G through I). LGALS3BP also inhibited replication of multiple PRRSV strains, including HP-PRRSV JX, classic strains BJ-4 and CH-1R, and NADC30-like strain HNhx, as shown by RT-qPCR (Fig. 2J). The antiviral activity of LGALS3BP was validated in porcine alveolar macrophages (PAMs) using a recombinant lentivirus expressing LGALS3BP. Following PRRSV infection, RT-qPCR and Western blot analyses showed successful overexpression of LGALS3BP (Fig. 2K and M), which resulted in significant reductions in viral RNA (Fig. 2L), protein (Fig. 2M), and titer (Fig. 2N).

**Fig 3:**
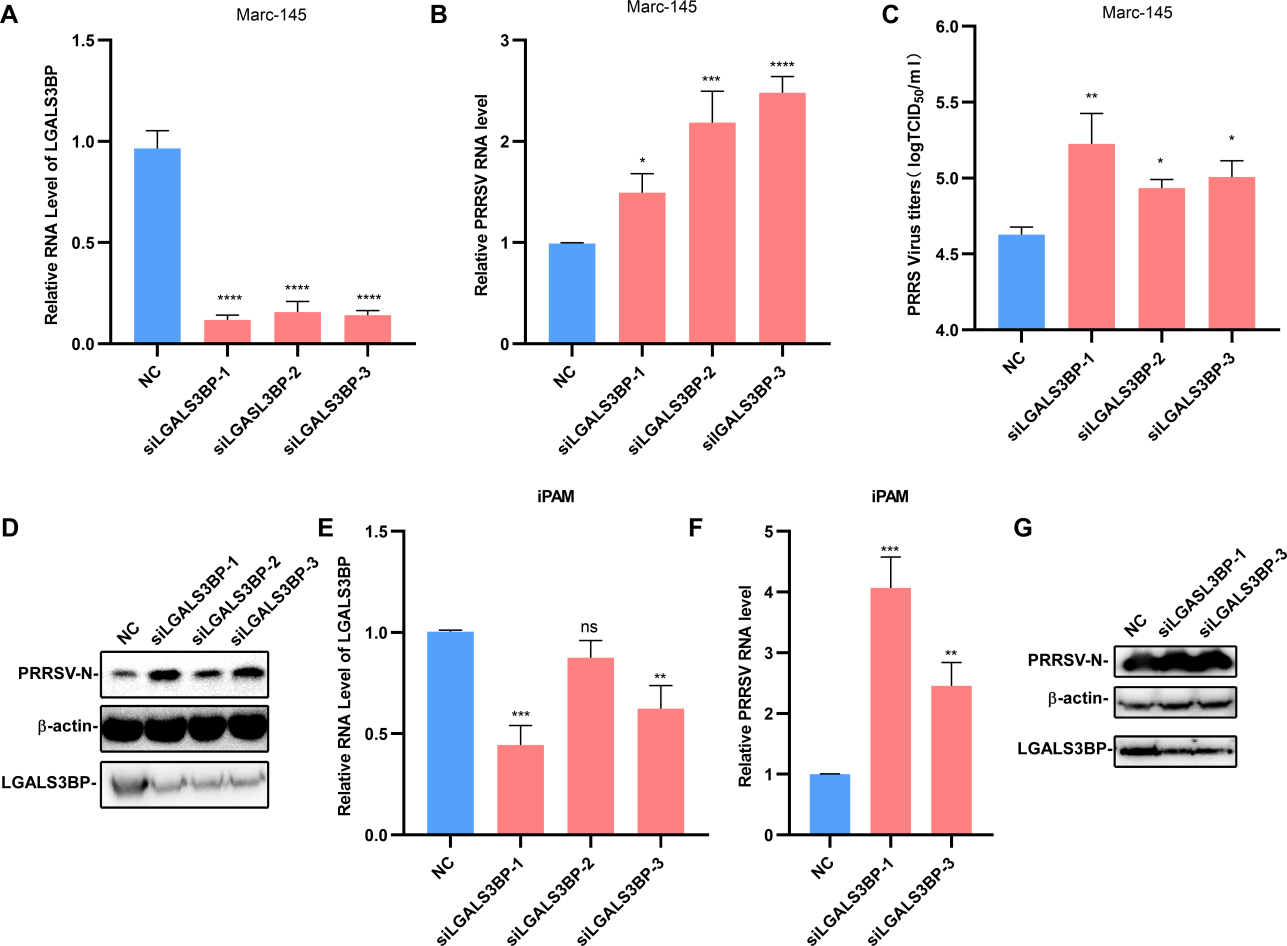
Knockdown of LGALS3BP enhances PRRSV infection. (**A–D**) Marc-145 cells were transfected with NC or siRNA-LGALS3BP for 24 h and then infected with PRRSV (MOI = 0.1) for another 24  h. The cell lysates and culture supernatants were collected to analyze *LGALS3BP* RNA levels (**A**), PRRSV RNA levels (**B**), viral titers (**C**), and PRRSV protein expression (**D**). (**E–G**) iPAMs were transfected with NC or siRNA-LGALS3BP for 24 h and then infected with PRRSV (MOI = 0.1) for another 24  h. The cell lysates and culture supernatants were collected to analyze LGALS3BP RNA levels (**E**), PRRSV RNA levels (**F**), and PRRSV protein expression (**G**). Data are presented as the mean ± SD from three independent experiments. Statistical significance is indicated as follows: *, *P* < 0.05; **, *P* < 0.01; ***, *P* < 0.001; ****, *P* < 0.0001).

Furthermore, knockdown of LGALS3BP using specific siRNAs in Marc-145 cells resulted in a significant increase in PRRSV RNA, protein, and titer levels (Fig. 3A through D). And the result of knocking down LGASL3BP in immortalized PAMs (iPAMs) is similar to that of Marc-145 cells (Fig. 3E through G). Taken together, these findings confirm LGALS3BP as a host factor with antiviral activity against PRRSV.

### LGALS3BP regulates PRRSV infection at the stage of replication

**Fig 4:**
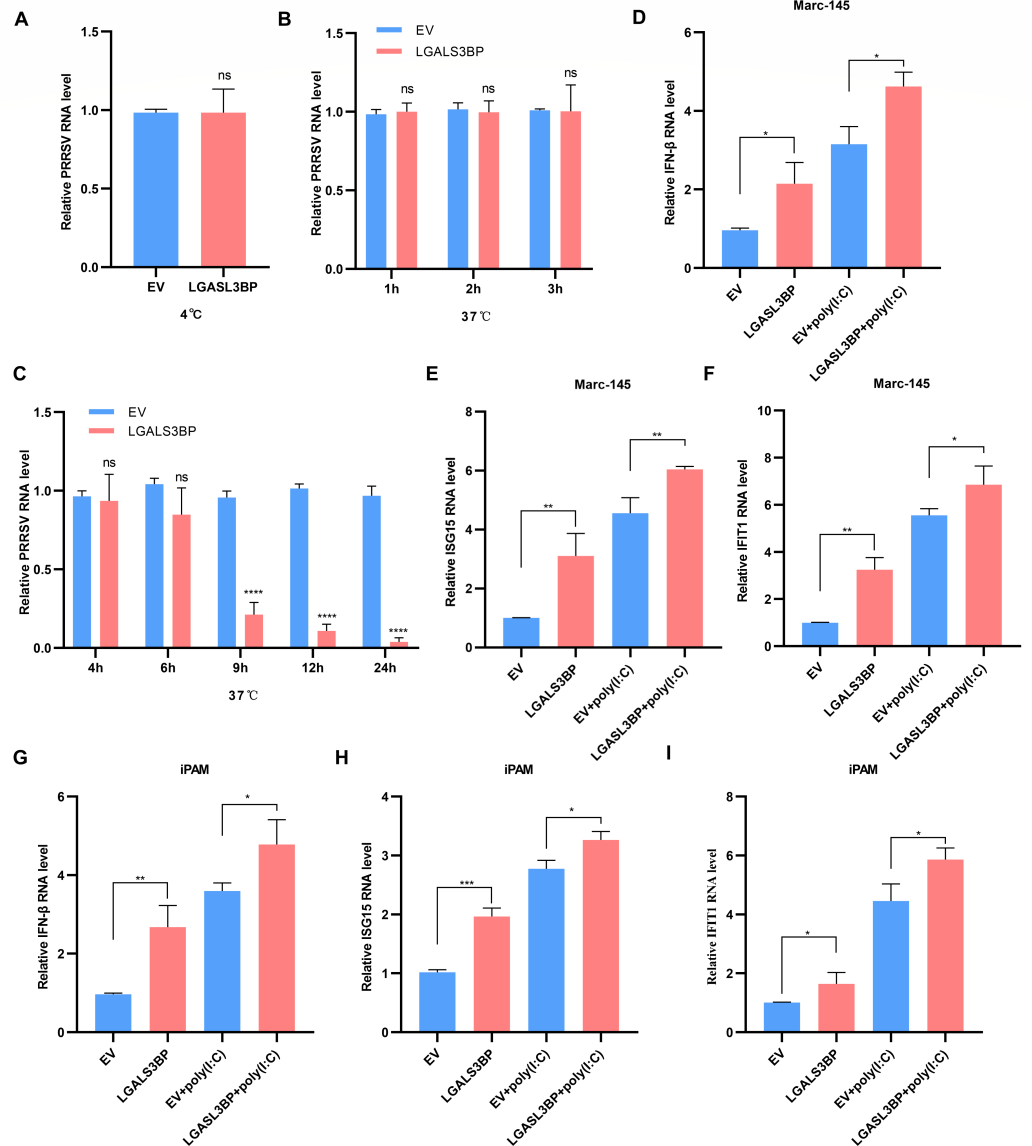
LGALS3BP regulates PRRSV infection at the stage of biosynthesis. (**A–C**) The overexpression of LGALS3BP does not affect virus attachment and entry. Marc-145 cells were transfected with EV or LGALS3BP for 24 h, incubated with PRRSV (0.1 MOI) at 4°C for 1 h (**A**), and then incubated at 37°C for 1 to 3 h (**B**) or 6 to 24 h (**C**). PRRSV RNA levels were determined by RT-qPCR. (**D–F**) Marc-145 cells were transfected for 24 h. The cell lysates were collected to analyze *IFN-β* (**D**), *ISG15* (**E**), and *IFIT1* (**F**) RNA levels. (**G–I**) iPAMs were transfected for 24 h. The RNA levels of *IFN-β* (**G**), *ISG15* (**H**), and *IFIT1* (**I**) were determined by RT-qPCR. Data are presented as the mean ± SD from three independent experiments. Statistical significance is indicated as follows: ns, *P* > 0.05; *, *P* < 0.05; **, *P* < 0.01.

To clarify LGALS3BP’s role in the PRRSV life cycle, Marc-145 cells were transfected with EV or LGALS3BP for 24 h, followed by PRRSV infection for 1 h at 4°C to allow viral attachment. After removing unbound virions, cells were shifted to 37°C and harvested at designated times for viral quantification. As shown in Fig. 4A and B, LGALS3BP overexpression did not affect PRRSV RNA levels at 1 hpi (4°C) or between 1 and 3 hpi (37°C), suggesting no impact on virus attachment or penetration. However, a significant reduction in viral RNA was observed at 9 hpi (Fig. 4C), indicating that LGALS3BP regulates PRRSV infection during the viral biosynthesis stage after entry into the host cell.

Inhibition of viral biosynthesis is commonly associated with the production of type I interferons (IFN-I, including *IFN-α/β*) or the activation of IFN-stimulated genes (ISGs). Therefore, we investigated the impact of LGALS3BP on IFN-I responses by transfecting Marc-145, HEK-293T-CD163 cells, and iPAMs with EV, LGALS3BP, and poly(I:C) via measuring *IFN-β* and ISG expression by RT-qPCR. The results revealed that overexpression of LGALS3BP significantly upregulated *IFN-β* and several ISGs, including IFN-stimulated gene 15 (*ISG15*) and IFN-induced protein with tetratricopeptide repeats 1 (*IFIT1*) (Fig. 4D through L). These results suggest that LGALS3BP enhances innate antiviral responses.

### LGALS3BP degrades nsp12 through the ubiquitin-proteasome pathway

**Fig 5:**
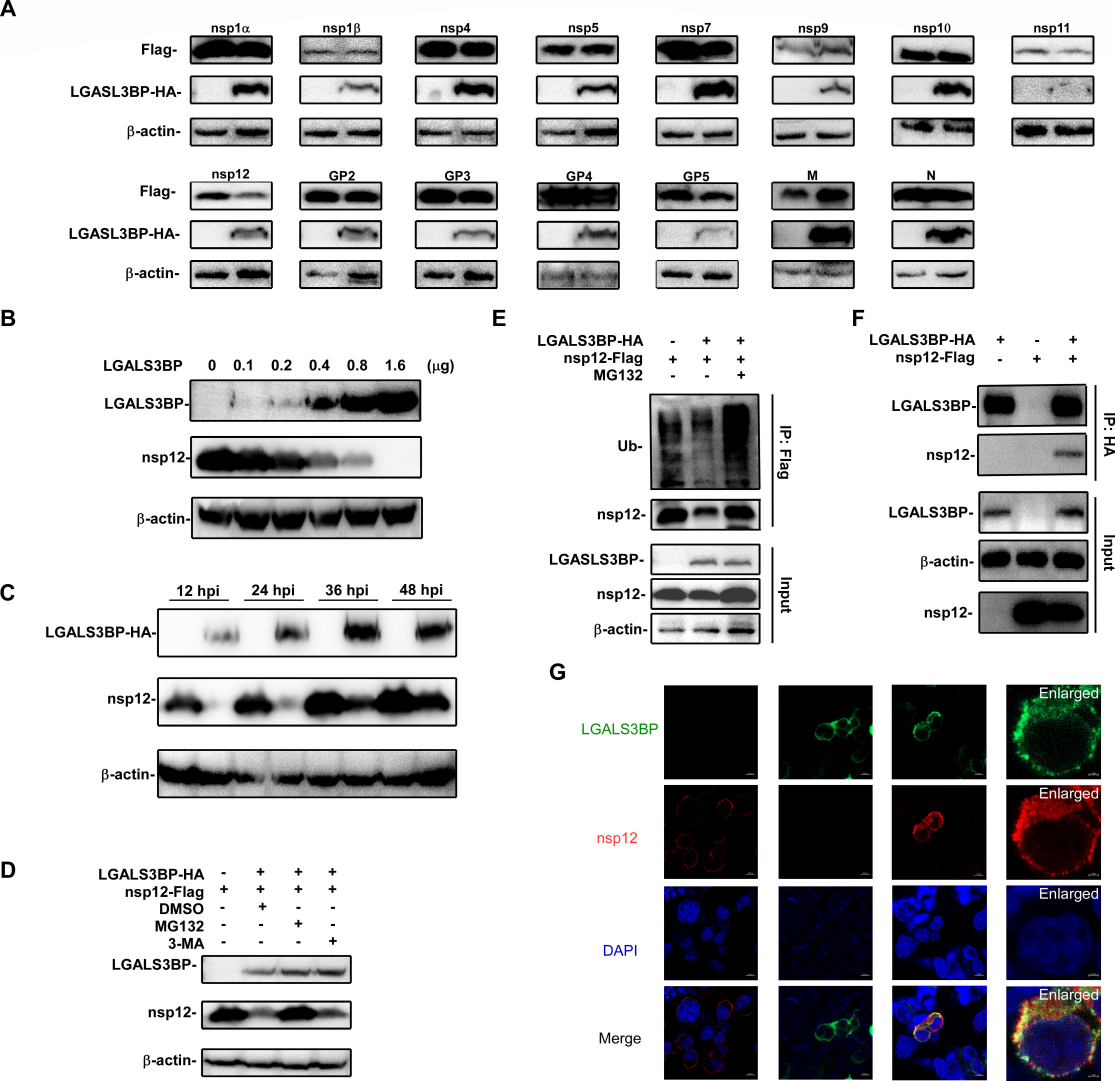
LGALS3BP specifically degrades the PRRSV nsp12. (**A**) HEK-293T cells were transfected with PRRSV nsps and LGALS3BP for 24 h. The cell lysates were collected to analyze PRRSV nsps protein expression. (**B**) HEK-293T cells were transfected with different doses of LGALS3BP (0.1, 0.2, 0.4, 0.8, and 1.6 µg) and nsp12 for 24 h. The cell lysates were collected to analyze nsp12 protein expression. (**C**) HEK-293T cells were transfected with LGALS3BP and nsp12 for 12, 24, 36, and 48 h. The cell lysates were collected to analyze nsp12 protein expression. (**D**) HEK-293T cells were transfected with nsp12 and LGALS3BP for 12 h, and then treated with MG132 (0.1 µM), or 3-methyladenine (3-MA) (1 mM) for 24 h. (**E**) HEK-293T cells were transfected with nsp12 and LGALS3BP for 12 h and treated with or without MG132 (0.1 µM) for 24 h. Cells were collected to analyze by Co-IP assay. (**F**) HEK-293T cells were transfected with LGALS3BP and nsp12 for 24 h. Cells were collected to analyze by Co-IP assay. (**G**) HEK-293T cells were transfected with nsp12 and LGALS3BP for 24 h, and double stained with a rabbit anti-HA antibody and a mouse anti-Flag antibody, followed by fluorescein Alexa Fluor-conjugated anti-rabbit IgG (green) and Alexa Fluor-conjugated anti-mouse IgG (red). Cell nuclei were counterstained with 1 µg/mL of 4’, 6’-diamidino-2-phenylindole.

Previous studies have demonstrated that LGALS3BP possesses ubiquitination activity and can promote the degradation of target proteins (22, 23). Based on this, we hypothesized that the inhibitory effect of LGALS3BP in PRRSV may also be mediated by proteolytic activity. To test this, we co-transfected expression plasmids for major PRRSV nonstructural or structural proteins with LGALS3BP into HEK-293T cells and analyzed viral protein expression at 24 h. The results showed that LGALS3BP specifically reduced the levels of nsp12. In contrast, other nonstructural proteins, including nsp1α, nsp1β, nsp4, nsp5, nsp7, nsp9, nsp10, and nsp11, and structural proteins, such as GP2(ORF2), GP3(ORF3), GP4(ORF4), GP5(ORF5), M(ORF6), and N(ORF7), showed no significant changes (Fig. 5A). A dose-dependent experiment revealed that increasing LGALS3BP levels led to a gradual decline in nsp12 expression (Fig. 5B). Concurrently, a time-course experiment exhibited a significant reduction in nsp12 expression as well (Fig. 5C).

To determine the degradation pathway, we co-transfected HEK-293T cells with LGALS3BP and nsp12 plasmids with MG132, a proteasome inhibitor, and 3-MA, an autophagy inhibitor. As shown in Fig. 5D, LGALS3BP-mediated nsp12 degradation was effectively blocked by MG132 but not by 3-MA, indicating that nsp12 degradation occurs through the ubiquitin-proteasome pathway. Furthermore, we observed a significant increase in nsp12 ubiquitination in the presence of LGALS3BP, further enhanced by MG132 (Fig. 5E). Co-immunoprecipitation (Co-IP) experiments exhibited an interaction between LGALS3BP and nsp12 (Fig. 5F), and laser confocal microscopy revealed their co-localization (Fig. 5G), suggesting that LGALS3BP facilitates nsp12 degradation through direct interaction.

### LGALS3BP mediates nsp12 degradation through the BACK domain

**Fig 6:**
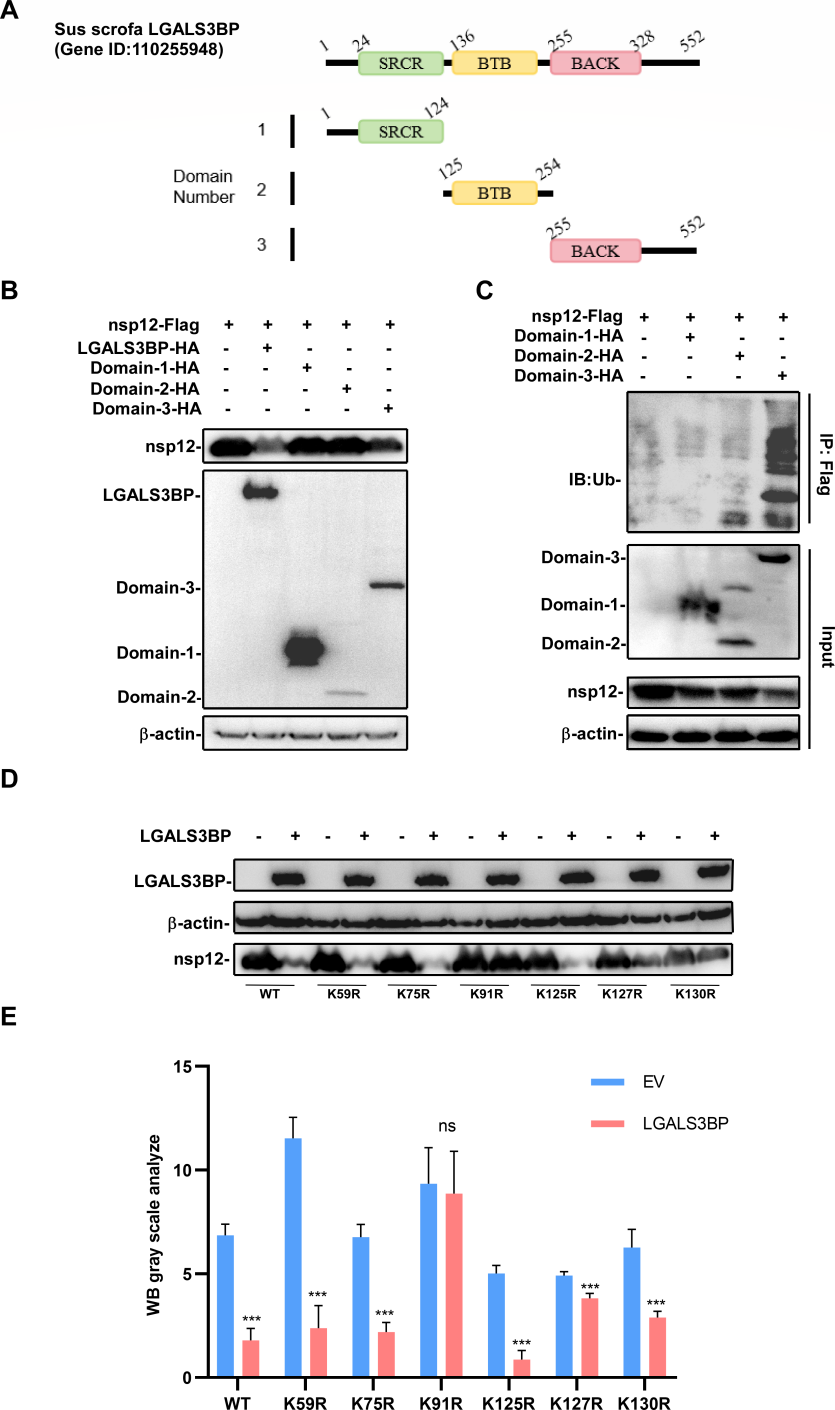
LGALS3BP degrades nsp12 through BACK domain. (**A**) Schematic diagram of truncated constructs of LGALS3BP. (**B**) HEK-293T cells were transfected with nsp12 and truncated mutant plasmids for 24 h. The cell lysates were collected to analyze nsp12 protein expression. (**C**) HEK-293T cells were transfected with LGALS3BP domain plasmids and nsp12 plasmids for 24 h. Cells were collected to analyze by Co-IP assay. (**D**) HEK-293T cells were transfected with LGALS3BP and nsp12 lysine site mutant plasmids for 24 h. The cell lysates were collected to analyze nsp12 protein expression. (**E**) Quantitative analysis results of the gray values in panel D. Statistical significance is indicated as follows: ***, *P* < 0.001.

We mapped the domains of LGALS3BP (SRCR, BTB, and BACK) and created truncation mutants (Fig. 6A). Co-transfection of these truncation mutants with nsp12 into HEK-293T cells demonstrated that the BACK domain significantly reduced nsp12 expression as determined by Western blot (Fig. 6B). Co-IP analysis showed that nsp12 ubiquitination was primarily dependent on the BACK domain, while the SRCR and BTB domains had no significant effects (Fig. 6C). To identify the critical ubiquitination site, we generated lysine (K)-to-arginine (R) mutants within the 154 amino acids of nsp12 (K59R, K75R, K91R, K125R, K127R, K130R) (Fig. 6D). Western blot analysis showed that the K91R mutant was resistant to LGALS3BP-mediated degradation, implicating that lysine 91 (K91) is the key ubiquitination site (Fig. 6D and E). These findings demonstrate that LGALS3BP mediates nsp12 degradation through BACK domain-dependent ubiquitination at the K91 residue.

### LGALS3BP mediates nsp12 degradation by recruiting the Cullin3 E3 ubiquitin ligase

**Fig 7:**
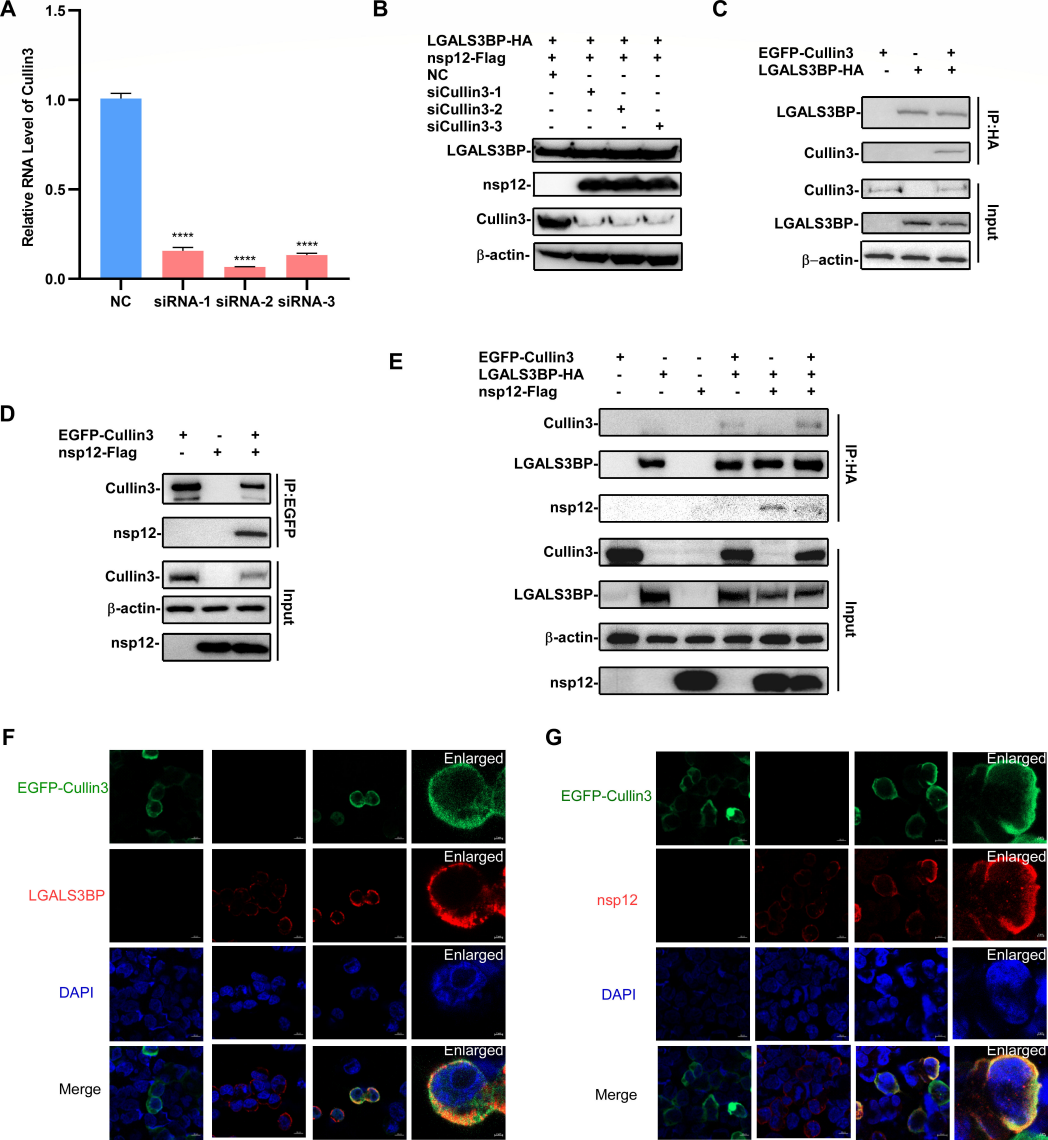
LGALS3BP degrades nsp12 by recruiting the Cullin3 E3 ubiquitin ligase. (**A**) HEK-293T cells were transfected with Cullin3-specific siRNAs for 24 h, cells were collected to analyze the Cullin3 mRNA levels by RT-qPCR. (**B**) HEK-293T cells were transfected with Cullin3-specific siRNA for 24 hours, followed by co-transfection with LGALS3BP and nsp12 plasmids for another 24 hours. The cell lysates were collected to analyze nsp12 protein expression. (**C and D**) HEK-293T cells were transfected with Cullin3 and LGALS3BP (**C**) /nsp12 (**D**) plasmids for 36 h. Cells were collected to analyze by Co-IP assay. (**E**) HEK-293T cells were transfected for 12 h, then treated with MG132 (0.1 µM) or DMSO for another 24 h. Cells were collected to analyze by Co-IP assay. (**F and G**) HEK-293T cells were transfected and stained with mouse anti-HA (**F**) antibody or mouse anti-Flag (**G**) antibody, followed by Alexa Fluor-conjugated anti-mouse IgG (red). Cell nuclei were counterstained with 1 µg/mL of 4′,6′-diamidino-2-phenylindole. Statistical significance was determined by one-way ANOVA (****, *P* < 0.0001).

The BACK domain of BTB-BACK-kelch proteins, such as KEAP1 (Kelch-like ECH-associated protein 1), stabilizes interactions with Cullin3 (24–28), facilitating the formation of the Cullin3-RING E3 ligase complex, which targets specific substrates for ubiquitination and proteasome degradation. Based on this, we hypothesized that LGALS3BP recruits Cullin3 to degrade nsp12 via its BACK domain. To test this, we knocked down Cullin3 using specific siRNA, which significantly attenuated LGALS3BP-mediated nsp12 degradation (Fig. 7A and B). Co-IP analysis confirmed interactions between LGALS3BP and Cullin3 (Fig. 7C), as well as between Cullin3 and nsp12 (Fig. 7D). Further Co-IP of co-transfected LGALS3BP, Cullin3, and nsp12 revealed that LGALS3BP pulled down both nsp12 and Cullin3, indicating the formation of ternary complexes (Fig. 7E). Confocal microscopy analysis also validated the co-localization of Cullin3 with both LGALS3BP and nsp12 (Fig. 7F through G). Collectively, these findings identify Cullin3 as the key E3 ubiquitin ligase responsible for LGALS3BP-driven nsp12 ubiquitination and degradation, providing novel insights into the antiviral mechanisms of LGALS3BP in PRRSV infection.

### LGALS3BP inhibits the synthesis of PRRSV subgenomic RNA

**Fig 8:**
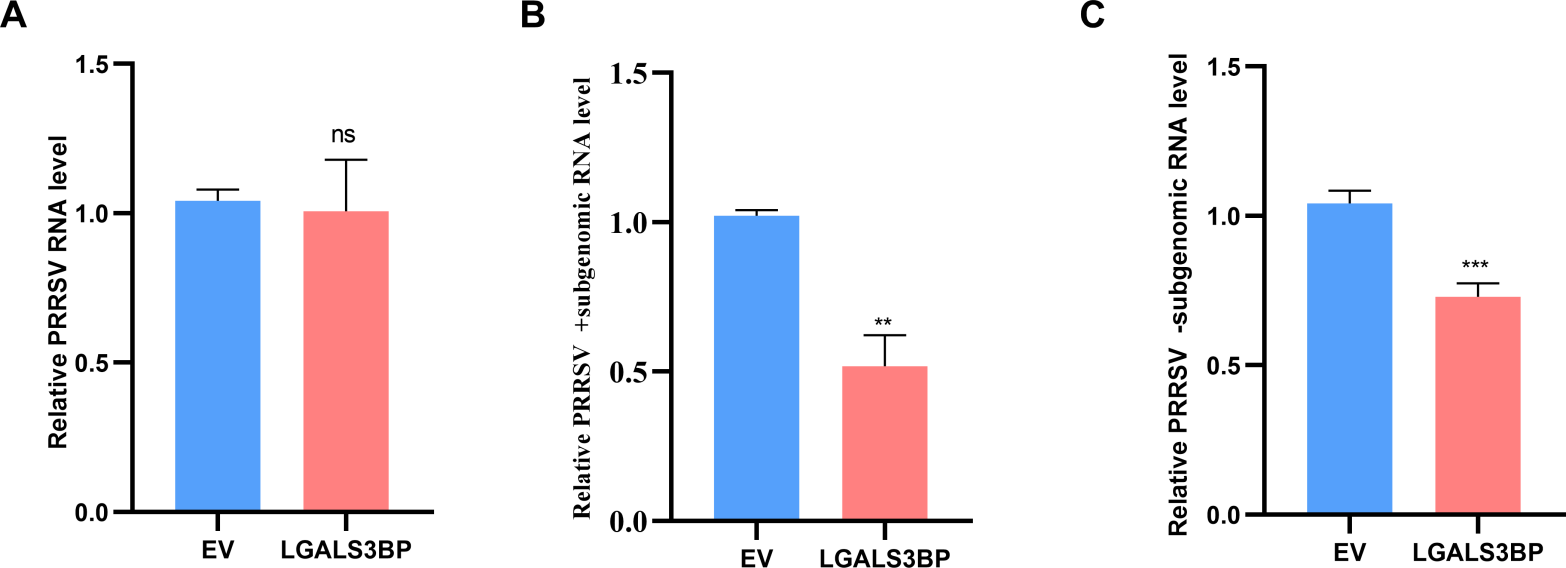
LGALS3BP inhibits the synthesis of PRRSV subgenomic RNA. (**A–C**) Marc-145 cells were transfected with LGALS3BP or EV for 24 h, followed by infection with PRRSV (0.1 MOI) for 4 h. Cells were collected to analyze PRRSV genomic RNA levels (**A**), PRRSV plus-strand subgenomic RNA levels (**B**), and PRRSV minus-strand subgenomic RNA levels (**C**). Data are presented as the mean ± SD from three independent experiments. ns, *P* > 0.05; **, *P* < 0.01, ***, *P* < 0.001. The *P* value was calculated using a Student’s *t*-test (two-tailed).

PRRSV nsp12 has been reported to play a key role in the synthesis of viral subgenomic mRNA (14). To determine whether LGALS3BP-mediated nsp12 degradation affects subgenomic mRNA synthesis, Marc-145 cells were transfected with LGALS3BP plasmids for 24 h and then infected with PRRSV (0.1 MOI) for 4 h. Cells were harvested to analyze viral RNA levels. Reverse transcription was performed using universal primers (for total viral RNA) or strand-specific primers (for subgenomic mRNA), followed by qPCR to quantify viral genomic RNA, plus- and minus-strand subgenomic mRNA levels. The results revealed that LGALS3BP significantly reduced subgenomic mRNA production before impacting viral genomic RNA levels (Fig. 8A through C), indicating that LGALS3BP inhibits viral replication by blocking subgenomic mRNA synthesis.

## DISCUSSION

The genetic, pathogenic, and antigenic variability of PRRSV strains remains a major challenge to global swine health and sustainable pork production (29, 30), underscoring the need for effective antiviral strategies. In this study, we identify LGALS3BP as a potent antiviral host factor that inhibits PRRSV infection. We demonstrate that LGALS3BP targets the viral non-structural protein nsp12, a critical component of the replication-transcription complex, leading to its proteasomal degradation. Our results show that overexpression of LGALS3BP significantly suppresses PRRSV replication, whereas its knockdown enhances viral replication. Furthermore, LGALS3BP enhances antiviral innate immune responses by upregulating IFN-β and ISGs. Together, these findings suggest that LGALS3BP restricts PRRSV through direct degradation of nsp12 and modulation of host immune responses, making it a promising therapeutic target for PRRSV control.

Our findings align with previous studies showing that LGALS3BP is upregulated during viral infections such as HIV, herpesvirus, influenza A virus, vesicular stomatitis virus, and severe acute respiratory syndrome coronavirus 2 (SARS-CoV-2), where it modulates antiviral immunity (15, 18, 19, 31, 32). In these cases, LGALS3BP has been linked to type I interferon responses. However, our study uniquely highlights LGALS3BP’s direct role in PRRSV inhibition through the degradation of nsp12. To our knowledge, this is the first report of LGALS3BP targeting a key viral replicase protein in a nidovirus like PRRSV. This contrasts with findings in SARS-CoV-2, where LGALS3BP’s antiviral activity appears to be mainly immune-modulatory, without direct degradation of viral replication proteins. This distinction underscores the unique antiviral mechanism of LGALS3BP in PRRSV infection.

The distinct antiviral mechanisms of LGALS3BP in PRRSV and other viruses highlight its versatile role in host defense. Although SARS-CoV-2 and PRRSV both belong to the *Nidovirales* order, our study demonstrates that LGALS3BP directly targets nsp12, a viral enzyme responsible for subgenomic RNA synthesis. In contrast, LGALS3BP’s antiviral effect in SARS-CoV-2 is primarily immune-modulatory. We hypothesize that these two mechanisms work in parallel to restrict PRRSV replication: (i) degradation of nsp12 via the ubiquitin-proteasome pathway and (ii) upregulation of IFN-β and ISGs; however, future studies are needed to dissect their interplay. The dual activity of LGALS3BP suggests it acts both as a direct viral suppressor and as an enhancer of antiviral immunity, providing a robust defense against PRRSV.

Initially identified for its role in cancer development, LGALS3BP has emerged as a potential therapeutic target (33, 34). Our findings show that LGALS3BP significantly upregulates IFN-β and ISGs, including ISG15 and IFIT1, in addition to its direct antiviral effect. This suggests that LGALS3BP not only inhibits viral replication but also enhances the host’s innate immune response. While this immune-modulatory role appears to be part of a general antiviral mechanism (35), the specific degradation of PRRSV nsp12 suggests that LGALS3BP has a targeted, virus-specific antiviral effect. This combination of immune regulation and viral protein degradation makes LGALS3BP an effective host factor against PRRSV and potentially other viral infections.

LGALS3BP, a multifunctional glycoprotein featuring SRCR, BTB/POZ, and BACK domains, participates in diverse biological processes (36–39). While homologous domains in other proteins have been characterized, the precise roles of LGALS3BP’s individual domains remain incompletely understood. The SRCR domain, a constituent of the SRCR protein receptor superfamily, mediates protein-ligand interactions (40). The BTB/POZ domain, prevalent in adaptor proteins, governs transcription, cytoskeletal dynamics, and ubiquitination (15). The BACK domain, conserved in BTB-kelch proteins, facilitates E3 ubiquitin ligase assembly (28, 41–43). Although LGALS3BP induces E-cadherin downregulation (23), the domain(s) responsible for this activity have yet to be identified. Here, we show that LGALS3BP mediates nsp12 degradation by recruiting Cullin3, an E3 ubiquitin ligase, via its BACK domain. Specifically, LGALS3BP forms a ternary complex with Cullin3 and nsp12, promoting K91-specific ubiquitination of nsp12 and subsequent proteasomal degradation. Co-IP and confocal microscopy confirmed the direct interactions among these proteins, while Cullin3 knockdown abolished LGALS3BP-dependent nsp12 degradation, establishing Cullin3 as essential for this process.

Nsp12, a highly conserved component of the RTC, is essential for PRRSV subgenomic RNA synthesis (44–47). Additionally, nsp12 is often targeted by the host restriction factors, such as PSMB1, RNF114, and GAL3 (48–50), underscoring its vulnerability as a viral dependency factor. We identified K91 in nsp12 as a conserved ubiquitination site across PRRSV strains, linking LGALS3BP-mediated ubiquitination to antiviral activity. These findings provide a novel mechanism by which LGALS3BP restricts PRRSV replication and highlight the ubiquitin-proteasome system as a key regulator of viral pathogenesis.

While our study provides compelling *in vitro* evidence for the antiviral activity of LGALS3BP, the findings should be validated in *in vivo* models. As PRRSV infects PAMs *in vivo*, future studies using PRRSV-infected pigs are needed to determine the therapeutic potential of LGALS3BP. These studies should assess viral loads, immune responses, and clinical outcomes to evaluate the efficacy of LGALS3BP in a more physiologically relevant system. Furthermore, future work should investigate whether LGALS3BP can be administered as a therapeutic agent to reduce PRRSV replication and improve disease outcomes in swine.

**Fig 9:**
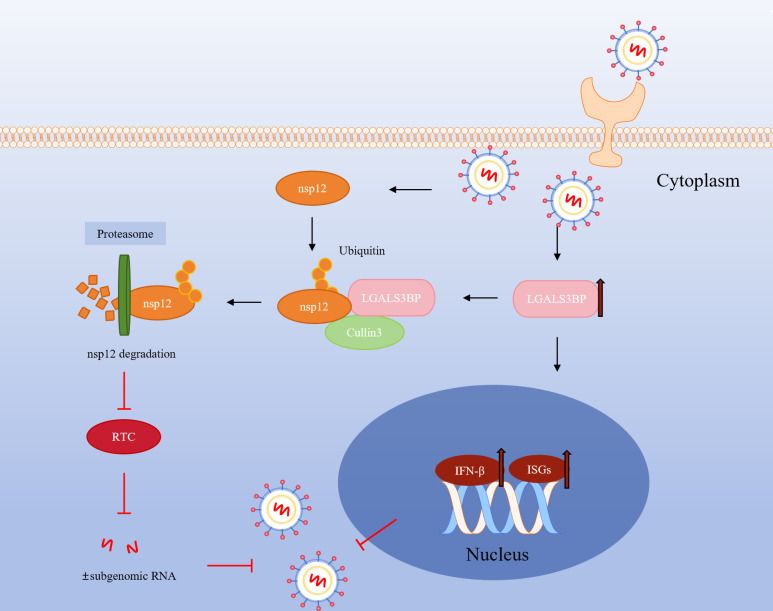
Schematic diagram of the inhibitory effect of LGALS3BP on PRRSV infection.

In conclusion, our study identifies LGALS3BP as a critical antiviral host factor that suppresses PRRSV infection by directly targeting nsp12 and modulating immune responses (Fig. 9). This dual mechanism, degradation of a viral protein and activation of antiviral immunity, suggests that LGALS3BP could serve as a novel therapeutic target for PRRSV control. Given its broad-spectrum antiviral activity, LGALS3BP holds promise as a candidate for host-directed therapies, not only for PRRSV but potentially for other viral infections as well.

## MATERIALS AND METHODS

### Cells and viruses

Marc-145 and HEK-293T cell lines were maintained in Dulbecco’s Modified Eagle Medium (DMEM; Gibco) supplemented with 10% heat-inactivated fetal bovine serum (FBS; Clarkbio) and 1% penicillin-streptomycin (Beyotime Biotechnology, China). Primary PAMs were maintained in our laboratory, having been isolated from the lungs of 5-week-old specific-pathogen-free Large White pigs. The PRRSV-susceptible iPAMs, developed by introduction of SV40 large T antigen, were kindly provided by Dr. Yan-dong Tang at Harbin veterinary research institute (51). Both PAMs and iPAMs were cultured in RPMI-1640 medium (Gibco) containing 10% FBS, 1% antibiotics. All cell cultures were incubated at 37°C in a humidified atmosphere with 5% CO₂.

The PRRSV strains employed in this study, including HuN4 (GenBank: EF635006), JX (GenBank: JX317649.1), BJ-4 (GenBank: AF331831.1), HNhx (GenBank: KX766379.1), and CH-1R (GenBank: EU807840), were preserved in our laboratory. The HuN4 strain served as the primary viral model throughout this investigation. Virus propagation and titration were performed in Marc-145 cells following standard protocols (52).

### Lentiviral preparation

Lentiviral production was performed as previously described with minor modifications (53). Briefly, HEK-293T cells were co-transfected with the lentiviral expression plasmid pLVX-IRES-ZsGreen1/LGALS3BP and the packaging plasmids psPAX2 and pMD2.G at a 3:2:1 mass ratio. Viral supernatants were collected at 48 and 72 h post-transfection, centrifuged at 4,000 × *g* for 5 min (4°C) to remove cell debris, and subsequently filtered through 0.45-µm PVDF membranes (Millipore) to obtain the clarified lentiviral stock. The harvested lentivirus was titrated in HEK293T cells with serial 10-fold dilution in the presence of 6 µg/mL polybrene. After 3–4 days of incubation, fluorescent cells from the last two dilution gradients were counted, and viral titer was calculated using the following formula: TU/mL = (X  +  Y  ×  10) × dilution factor × 10, where X and Y denote the number of fluorescent wells at each respective dilution. For transduction experiments, the PAMs were exposed to lentivirus at a MOI of 5, achieving greater than 50% fluorescent-positive cells.

### Antibodies and reagents

The anti-PRRSV-N antibody was prepared in our laboratory. Commercial antibodies included anti-Flag (MA1-91878), anti-HA (26183), goat anti-mouse IgG (H+L) cross-adsorbed secondary antibody (G-21040), and goat anti-rabbit IgG (H+L) cross-adsorbed secondary antibody (G-21234) from Invitrogen; anti-β-actin (A2228) from Sigma-Aldrich; BeyoMag Protein G beads (P2105), Western/IP lysis buffer (P0013), 4′,6′-diamidino-2-phenylindole (DAPI, C1002), Alexa Fluor 555-labeled donkey anti-rabbit IgG (H+L) (A0452), and Alexa Fluor 488-labeled goat anti-mouse IgG (H+L) (A0428) from Beyotime Biotechnology. Pharmacological agents MG132 (HY-13259) and 3-methyladenine (3-MA) (HY-19312) were purchased from MedChemExpress (MCE). The porcine LGALS3BP ELISA kit was sourced from Shanghai Coibo Biotechnology.

### Construction of plasmids

PrimeSTAR HS DNA polymerase was used to amplify the LGALS3BP gene (GenBank: XM_021066516.1) from PAMs’ cDNA before cloning it into the pCAGGS expression vector with a C-terminal-HA. Similarly, the PRRSV nsps’ genes were amplified from cDNA of PRRSV HuN4-infected Marc-145 cells using PrimeSTAR HS DNA polymerase and cloned into pCAGGS with a C-terminal-Flag. Plasmids for Cullin3 were purchased from Miaoling Biotechnology (Wuhan, China).

### RNA-mediated interference (RNAi)

Small-interfering RNAs (siRNAs) against LGALS3BP and Cullin3 were synthesized by GenePharma (Shanghai, China). Marc-145 or HEK-293T cells were transfected with 50 nM of either siRNA-LGALS3BP or siRNA-Cullin3 using the siRNA-MATE plus reagent (Genepharma) following the recommended procedure. The siRNA sequences are listed in Table 1.

**TABLE 1:** Sequences of siRNAs used in this study

siRNA name	siRNA sense sequence (5′−3′)	siRNA antisense sequence (5′−3′)
siLGALS3BP-Monkey-1	GAACGAUGGUGACAUGCGGTT	CCGCAUGUCACCAUCGUUCTT
siLGALS3BP-Monkey-2	AAGGUCCGCUUCCCUAUGATT	UCAUAGGGAAGCGGACCUUTT
siLGALS3BP-Monkey-3	CCUACGAAAACAAAGCCCUTT	AGGGCUUUGUUUUCGUAGGTT
siLGALS3BP-Porcine-1	CGCGUAGAGAUCUUCUACATT	UGUAGAAGAUCUCUACGCGTT
siLGALS3BP-Porcine-2	GUGAUCUGCACCAAAGAAATT	UUUCUUUGGUGCAGAUCACTT
siLGALS3BP-Porcine-3	GAGACUACAUCAGGUACCUTT	AGGUACCUGAUGUAGUCUCTT
siCullin3-1	GCGAGAAGAUGUACUAAAUTT	AUUUAGUACAUCUUCUCGCTT
siCullin3-2	GGCAAACUCUAUUGGAUAUTT	AUAUCCAAUAGAGUUUGCCTT
siCullin3-3	CUGCUAUAGUGCGGAUAAUTT	AUUAUCCGCACUAUAGCAGTT

### Reverse transcription-quantitative PCR

Total RNA was extracted from cells, using RNA extraction kits (BioFlux, China), and reverse transcribed into cDNA using PrimeScript RT reagent Kit (Takara, Japan) according to the manufacturer’s instructions. The ChamQ Universal SYBR qPCR Master Mix (Vazyme, China) was used to quantify mRNA levels. The primer sequences are listed in Table 2. Fold changes were determined using the cycle threshold (ΔΔCT) method.

**TABLE 2:** Sequences of primers used in this study

Primer name	Sequence (5′−3′)
PRRSV-N-F	AGATCATCGCCCAACAAAAC
PRRSV-N-R	GACACAATTGCCGCTCACTA
Porcine-β-actin-F	CTTCCTGGGCATGGAGTCC
Porcine-β-actin-R	GGCGCGATGATCTTGATCTTC
Monkey-β-actin-F	ATCGTGCGTGACATTAAG
Monkey-β-actin-R	ATTGCCAATGGTGATGAC
LGALS3BP-F	GGCCCCTCTATGGCTCCTAT
LGALS3BP-R	CATGTCACCGTCTTTCACGC
Porcine-ISG15-F	CAGAGACCCACTGAGCATCC
Porcine-ISG15-R	GCGTCAGCCAGACCTCATAG
Porcine-IFNB-F	TGCATCCTCCAAATCGCTCT
Porcine-IFNB-R	ATTGAGGAGTCCCAGGCAAC
Porcine-IFIT1-F	TCCGACACGCAGTCAAGTTT
Porcine-IFIT1-R	TGTAGCAAAGCCCTGTCTGG
Monkey-IFNB-F	TGCTCTCCTGTTGTGCTTCTC
Monkey-IFNB-R	CTGCGGCTGCTTAATTTCCTC
Monkey-ISG15-F	CACCGTGTTCATGAATCTGC
Monkey-ISG15-R	CTTTATTTCCGGCCCTTGAT
Monkey-IFIT1-F	GGCTACAAAAGGGCAGCCTA
Monkey-IFIT1-R	GCCAGGTCTAGATGAGCCAC
Cullin3-F	CGAATCTGAGCAAAGGCACG
Cullin3-R	TCCATGGTCATCGGAAAGGC
PRRSV-sgmRNA-F	GCCCAAAACTTGCTGCACG
PRRSV-sgmRNA-R	GACACAATTGCCGCTCACTA

### Transfection and Western blotting

The cells were transfected with indicated plasmids using Lipofectamine 2000 (Invitrogen). After 24 h, the cells were collected and lysed in Western and IP lysis buffer (Beyotime) with 1% PMSF (Beyotime). The cell supernatants were collected after centrifugation for 10 min at 12,000 × *g* and mixed with SDS-PAGE loading buffer, followed by boiling at 100°C for 10 min. The samples were separated with SDS-PAGE and then transferred to polyvinylidene difluoride (PVDF) membranes (Merck Millipore, USA), which were blocked in 5% skim milk and incubated with the indicated primary and secondary antibodies.

### Co-immunoprecipitation assay

After 24 h of transfection, cells were collected and lysed in Western and IP lysis buffer supplemented with 1% PMSF. The cell supernatants were collected after centrifugation for 10 min at 12,000 × *g*. A 100-µL aliquot of supernatant was taken as the input sample, mixed with SDS-PAGE loading buffer, and boiled at 100°C for 10 min. The remaining supernatant was incubated with monoclonal anti-Flag, anti-HA, or anti-GFP antibodies for 2 h. Following incubation, 20 µL Beyomag protein G beads was added, and the mixture was incubated for an additional 2 h. The beads were then washed five times with PBST, and the samples were analyzed by Western blotting.

### Confocal imaging

HEK-293T cells were co-transfected with indicated plasmids for 24 hpi. The cells were fixed in 4% paraformaldehyde for 30 min, permeabilized with 0.1% Triton X-100 for 15 min, and blocked with 3% bovine serum albumin for 1.5 h. The transfected cells were incubated with mouse anti-HA MAb and rabbit anti-Flag MAb for 1 h at RT and washed three times with PBST. The cells were then incubated at 37°C for 1 h with goat anti-mouse IgG (H+L) antibody conjugated with Alexa Fluor 488 and donkey anti-rabbit IgG (H+L) antibody labeled with Alexa Fluor 555. Finally, the cells were stained with 1 µg/mL of DAPI for 5 min and examined using a Zeiss confocal system.

### RT-qPCR of sgmRNA

Marc-145 cells were transfected with LGALS3BP plasmids for 24 h, followed by infection with PRRSV at 0.1 MOI. Cells were harvested at 4 hpi for RNA extraction. For +sgmRNA detection, cDNA was amplified with oligo-d(T). For -sgmRNA detection, cDNA was amplified using the primer: 5′-GTGTTGGCTCATGCCACGGC-3′. Strand-specific quantification of +sgmRNA and −sgmRNA was then conducted by qPCR using the primers provided in Table 1.

### Statistical analysis

Statistical analyses were carried out using Prism 8.0. Most experiments were conducted with at least three independent replicates. Data are presented as means ± standard errors (SD) from three or more independent experiments. One-way analysis of variance or Student’s *t*-test (two-tailed) was used for statistical comparisons. Significance levels were defined as: ns, *P* > 0.05; *, *P* < 0.05; **, *P* < 0.01; ***, *P* < 0.001; and ****, *P* < 0.0001.

## Data Availability

The RNA-seq data used in this study were deposited in the NCBI database with the accession number PRJNA997941.
